# Larval Pigmentation Reveals Environmental and Genetic Influences in Hybridizing Ambystoma Salamanders

**DOI:** 10.1002/ece3.71911

**Published:** 2025-08-03

**Authors:** Alfredo Ascanio, Victor Fitzgerald, Patrick Altomari, Jason T. Bracken, Tereza Jezkova

**Affiliations:** ^1^ Department of Biology Miami University Oxford Ohio USA

**Keywords:** Ambystoma, chromatophores, hybrid inferiority, hybridization, physiological response, ultraviolet radiation

## Abstract

Closely related species may produce hybrids, and these hybrids often display intermediate traits that can influence fitness and reproductive isolation. In this study, we examine *Ambystoma barbouri* and *Ambystoma texanum* , two sister salamander species that breed in contrasting aquatic habitats with differing levels of ultraviolet radiation (UVR) exposure. Although these species can hybridize, it remains unclear whether hybrid pigmentation responses to UVR confer an advantage, a disadvantage, or are simply intermediate between parental forms. Pigmentation in salamanders is regulated by chromatophores, melanophores, and xanthophores, which help mitigate UVR‐induced damage. Given previous knowledge on the species, habitats, and behaviors, we hypothesized that: (1) *A. barbouri* would show stronger UVR‐induced pigmentation (i.e., greater darkening) than *A. texanum* , due to its exposure to clearer, shallower streams; (2) hybrids would exhibit intermediate responses; and (3) xanthophore coverage would remain stable or decrease under UVR as melanophores expand. To test these hypotheses, we conducted a fully factorial breeding experiment using pure and reciprocal hybrid crosses, exposing larvae to four UVR durations (0, 1, 4, and 12 h). We quantified skin darkness and chromatophore‐specific pigmentation using standardized digital image analysis, Bayesian beta regression, and Bayesian compositional regression. Our results supported all three hypotheses. *A. barbouri* showed greater darkening under UVR exposure than *A. texanum* , and hybrids exhibited intermediate responses, but with a greater similarity toward their maternal phenotype. Xanthophore coverage remained stable or declined with increasing UVR exposure, evidencing that their main purpose is different from UVR protection. These findings raise the possibility that hybrids could experience reduced performance if intermediate pigmentation is suboptimal, but that maternal effects may offset some of these disadvantages.

## Graphical abstract

We investigated UV‐induced pigmentation responses in *Ambystoma barbouri* , *A. texanum* , and their hybrids, finding that *A. barbouri* larvae darken more strongly than *A. texanum* , with hybrids showing intermediate phenotypes closer to their maternal lineage. These patterns suggest potential maternal effects influencing pigmentation plasticity, which may shape hybrid performance and species boundaries in heterogeneous environments. Our results highlight how environmental gradients like UVR exposure can impact hybrid phenotypes, although direct fitness consequences remain to be tested.

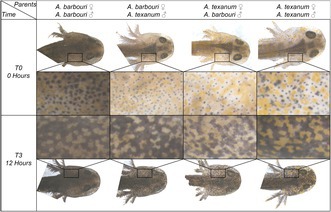

## 1 Introduction

When individuals from closely related species have different phenotypes, their hybrids may show intermediate characteristics (Adavoudi and Pilot 2021; McCarthy 2006; Thompson et al. 2021). If these parental phenotypes have been under different selective pressures in their respective environments, hybrid inferiority can occur as a mechanism of extrinsic postzygotic isolation (Coyne and Orr 2004; Rometsch et al. 2020). As a result, hybrid traits will not be well adapted to either parental habitat, resulting in lower fitness when compared to parental traits (Rundle and Nosil 2005). Under this scenario, we expect introgression between parental species to be limited, contributing to the separation between species (Coughlan and Matute 2020). Conversely, hybrids could display increased fitness when compared to their parental species, a pattern known as heterosis (Eizadshenass and Singh 2015; Videvall et al. 2016). Additionally, in some cases, F1 hybrids show heterosis due to increased genetic diversity, while their F2 and subsequent generations suffer from hybrid inferiority (Johnson et al. 2010). Therefore, knowing what phenotypic responses differ under environmental pressures in naturally hybridizing species can help us understand patterns of speciation and introgression.

Ultraviolet radiation (UVR) is an environmental factor that induces strong selective pressures in a variety of systems, often with physiological and behavioral trade‐offs related to cryptic coloration, feeding opportunity, predator avoidance, and UVR avoidance (Garcia et al. 2004, 2009b; Lee and Hansson 2021). The degree of response to UVR exposure can tell us much about the ecology and selective pressures that species face. UVR is beneficial in small doses and is critical for metabolic function (Antwis and Browne 2009; Bouillon and Suda 2014; Holick 1992; Lundsgaard et al. 2021; Pahkala et al. 2003; Tapley et al. 2015). Conversely, at higher doses, UVR can cause damage to DNA and induce death and malformation in developing organisms and adults (Ankley et al. 2002; Häder et al. 2011; Kern et al. 2015; Tietge et al. 2001). However, protection from UVR associated risks can result in costly trade‐offs, decreasing immune response and activity rates, while increasing predation risk (Cramp et al. 2022; Garcia et al. 2009a; Gorokhova et al. 2013). Additionally, different selective pressures, such as predation risk and UVR exposure, can act in concert to favor similar phenotypic traits, for example, increasing melanin content to increase camouflage against a dark background (Liedtke et al. 2023) while increasing protection to UVR (Tang et al. 2025).

The methods that organisms use to protect themselves are varied, from pigmenting the skin with UVR absorbing or reflecting proteins to extensive behavioral modifications to avoid exposure (Blaustein and Belden 2003; Brozio et al. 2021; Demori et al. 2019; Rudh and Qvarnström 2013; Thurman et al. 2014). Both behavioral and physiological mechanisms increase energy utilization, and organisms that do not require UVR protection can invest more into antipredator coloration and behaviors (Stoehr 2006). Local adaptation to UVR exposure can be affected by the degree of introgression between differentially adapted populations (Zhang et al. 2016). In a hybridization event between two species with differing levels of UVR protection, it is necessary to assess hybrid responses and whether they can contribute or not to introgression between species (Macagno et al. 2021; McGee et al. 2015; Parris 2004).

The sister species *Ambystoma barbouri* (streamside salamander) and *A. texanum* (small‐mouthed salamander) present an interesting system to explore adaptation to UVR, and whether it could influence hybrid performance or contribute to reproductive barriers between taxa. Genetic and experimental data suggest that *A. barbouri* and *A. texanum* can hybridize, although the extent of hybridization in nature is not fully understood (Denton et al. 2014; Fitzgerald 2023). As the conservation status of *A. barbouri* is currently being assessed by the US Fish & Wildlife Service (Nail 2023), it is imperative to understand the potential ecological and physiological factors limiting the gene flow between these taxa. Both sister species are almost indistinguishable morphologically, but utilize very different environments for breeding (Kraus and Petranka 1989). Consequently, their larval habitats differ in the amount of UVR exposure (Garcia and Sih 2003; Storfer and Sih 1998). Specifically, *A. barbouri* adults migrate from underground refugia in the spring to first‐order stream pools, where they mate and lay their eggs, firmly attaching them to the undersides of limestone rocks (Petranka 1982). The streams preferred by *A. barbouri* are typically shallow and clear, with little refugia from sunlight (Garcia and Sih 2003). It has been shown that when placed under similar conditions, *A. barbouri* larvae seem to prefer shallower waters than *A. texanum* , with greater exposure to UVR (Garcia et al. 2004). On the contrary, *A. texanum* breeds in typical *Ambystoma* habitat, emerging in the spring and laying their eggs on vegetation within vernal pools (Kraus and Petranka 1989). The vernal pools used by *A. texanum* are typically located in forested areas, being dense in refugia and displaying high turbidity due to the high input of leaves. Leaves release organic matter in the form of dissolved and particulate organic matter (Bayarsaikhan et al. 2016), limiting penetration of light in the water column (Häder et al. 1998). Vernal pools used by *A. texanum* also tend to exceed the depth of pools within the shallow first‐and second‐order streams preferred by *A. barbouri* (Kraus and Petranka 1989). These differences in breeding habitat suggest marked differences in the UVR pressures these species may face, and this can be reflected in their pigmentation. Previous work has shown that both species have larvae that are highly plastic in their physiological responses to UVR. *Ambystoma barbouri* larvae tend to be darker and have a stronger darkening response to UVR than *A. texanum* (Garcia 2002; Garcia et al. 2003; Garcia and Sih 2003). Additionally, in *A. barbouri* , pressure to be protected against UVR appears to be stronger than the pressure to camouflage against fish predators (Garcia et al. 2004; Storfer et al. 1999). Contrary to this, it is expected that *A. texanum* is subject to lower UVR pressures, as less light penetrates deeply into vernal pools and no fish predators are present (Garcia et al. 2004). In this case, *A. texanum* may receive a benefit in development speed and size as a tradeoff for a lower response to UVR (Garcia et al. 2004). Overall, this serves as evidence that UVR can be a major pressure on developing *Ambystoma* larvae, and that hybrid larvae may be maladapted to their parental environments and suffer fitness consequences if they were to exhibit intermediate phenotypes.

We can study UVR response in *Ambystoma* spp. by observing light‐induced changes in pigmentation cover. Such pigments are synthesized and stored within chromatophores, and are a common way of preventing UVR penetration into the skin, reducing DNA damage (Armstrong et al. 2000; Gouveia et al. 2004; Hargis 1981). Chromatophore cells include melanophores and xanthophores, which produce different pigments related to UVR protection. Melanophores produce and contain melanins, dark pigments that have anti‐oxidant properties and provide protection against UV light (Meredith and Sarna 2006). These melanins can be either phaeomelanins or eumelanins, the latter being a black pigment that can absorb high energy photons with a wavelength of 240–330 nm corresponding to UVB radiation (Meredith and Sarna 2006; Tang et al. 2025), preventing further infiltration into skin (Armstrong et al. 2000). Melanophores in *Ambystoma* contain eumelanin (Frost et al. 1984), therefore providing UVB radiation protection to these animals. In amphibians, melanin cover increases during UVR exposure and may serve to prevent death in eggs and larvae (Blaustein and Belden 2003; Lesser et al. 2001). The change in melanin cover is mediated by the contraction and expansion of melanophore cells in response to UVR. On the other hand, xanthophores, which range from red to yellow, are typically associated with predation reduction or sexual selection, rather than UVR protection (Rudh and Qvarnström 2013). However, larval caudates display both pigments even in the absence of sexual selection or visual predators at early life stages (Olsson 1994). Currently, xanthophore‐related pigments are understudied in larval amphibians.

Here, we hypothesize that *A. barbouri* will display a stronger response to UVR exposure than *A. texanum* . If this is the case, we predict that the melanophore cover will increase more in *A. barbouri* than in *A. texanum* . Furthermore, considering that introgression has been observed in the wild, we hypothesize that hybrids between both species will show intermediate phenotypic responses to UVR. Whether these intermediate phenotypes are indicative of heterosis or hybrid inferiority depends on the strength of their response under UVR exposure. Though we do not measure fitness directly, we consider that increased phenotypic plasticity in hybrids after UVR exposure would be indicative of heterosis, while narrower ranges than the parental phenotypes would indicate hybrid inferiority. Lastly, if xanthophores do not play a role in UVR protection during the larval stage of these salamanders, we predict that xanthophore cover should remain somewhat constant or decrease as UVR exposure increases to leave space for the expanding melanophores.

## 2 Materials and Methods

### 2.1 Study Site, Salamander Collection and Breeding

#### 2.1.1 Sampling

**FIGURE 1:**
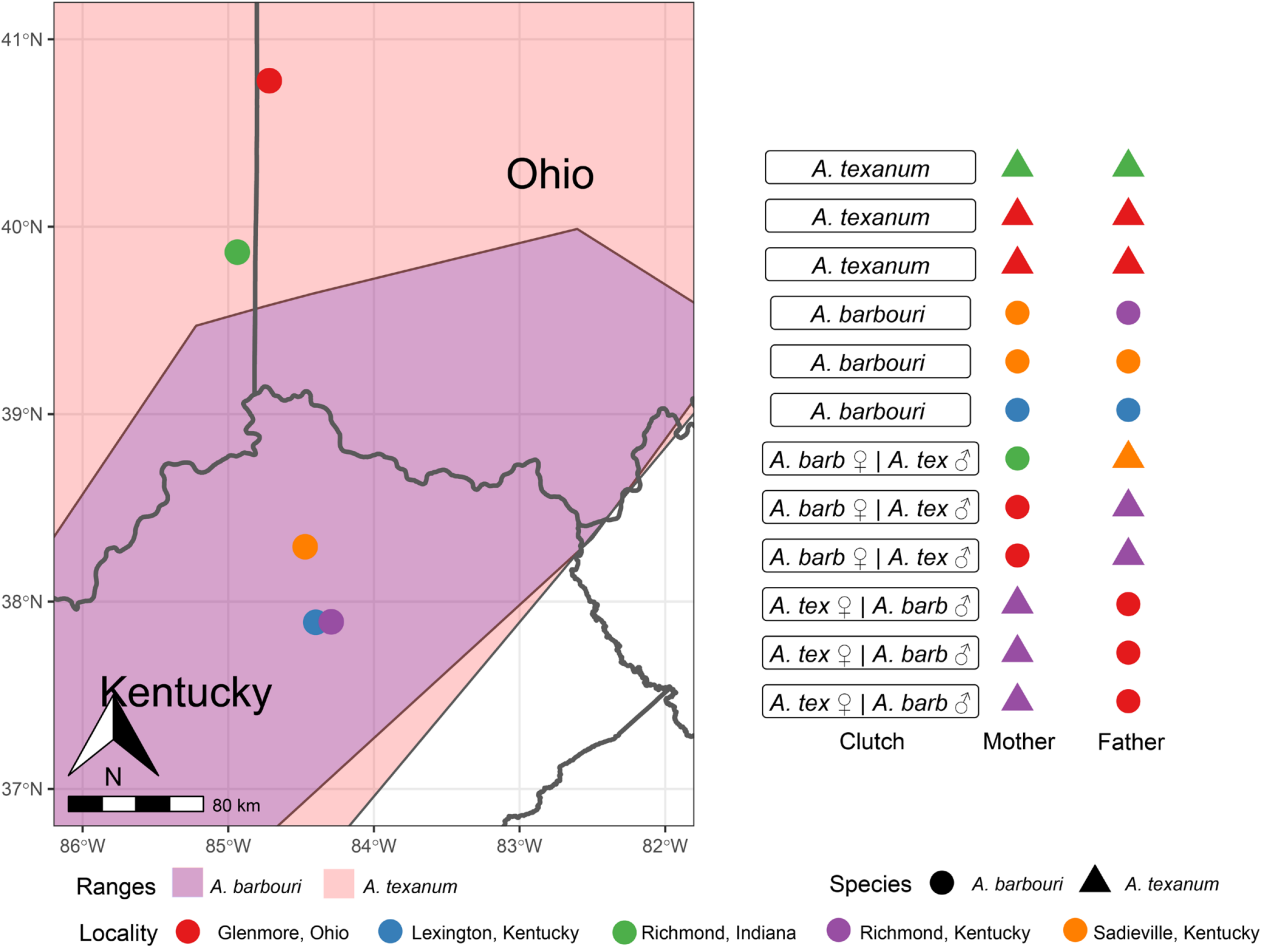
Study area showing localities where parental salamanders were captured and their respective pairings by species and location.

We collected adult salamanders from Ohio, Indiana, and Kentucky, choosing study sites for each species based on habitat type, either streams or vernal pools, for *A. barbouri* and *A. texanum* , respectively (Figure 1). We used 3 localities for *A. barbouri* exclusively from Kentucky and 2 localities for *A. texanum* from both Ohio and Indiana. As these species are suspected of hybridizing in the wild (Denton et al. 2014; Fitzgerald 2023), and to reduce the chance of acquiring individuals of mixed ancestry at the sampling localities, we chose sites away from the expected sympatric zone. Once collected, we transported adults back to laboratory‐aquaria filled with 54.5 L of dechlorinated water and 3.78 L of field site water. We placed adults in conspecific or heterospecific pairings within 48 h of their collection night. Both conspecific and heterospecific pairings readily bred and after 6 days we removed the adults from aquaria, while eggs remained until hatching. We supplemented eggs with additional aeration and otherwise left them undisturbed. Hatching occurred over a 10‐day period following the hatching of the first larvae. We removed larvae from the aquaria as they hatched and placed them into 13.6 L tubs filled with dechlorinated water and separated by genotype (parental pairing) and clutch. We then fed each larvae group with brine shrimp *ad libitum*. To reduce possible ontogenic effects on color response, all genotypes were exposed to UVR 27 days after the mean hatching date of the genotype.

### 2.2 Experimental Set‐Up

To test our hypothesis of differential pigmentation responses to UVR and determine potential hybrid effects alongside parental effects from maternal or paternal origins in this pair of *Ambystoma* salamanders, we performed a fully orthogonal experiment with three factors: exposure time, mother genotype, and father genotype. Exposure time was a fixed factor with 4 levels: 0 h, 1 h, 4 h, and 12 h. Mother and father genotypes were fixed factors with 2 levels each: *A. barbouri* and *A. texanum* . Genotypes for each larval salamander clutch were determined by conspecific and interspecific parental combinations of *A. barbouri* and *A. texanum* (Fitzgerald 2023). To assess potential intra‐genotype variations, three clutches per pairing were randomly selected. For each exposure time, 6 larvae per clutch were selected, giving a total of 288 larvae in our experiment. In addition to this, a 4 h temperature‐only treatment, simulating the same temperature increase reached by the 4 h UVR exposure treatment, was added to control for changes in coloration that could be due to the rise in temperature rather than the UVR exposure within the same time frame (Material S2).

To simulate high UVR we used a T8 ReptiSun 10.0 UVB bulb, which is typically used to supply UVR to captive amphibians and reptiles. We placed the larvae in petri dishes with a depth of water of 2.5 cm, and held the lightbulb 6 in. (15.24 cm) above them during the UVR treatment to establish high UVR exposure during our treatments. We used a BIC logger (BIC2104RL, Biospherical Instruments Inc., San Diego, CA) to determine dosages (kJ m^−2^) of 305 nm, 320 nm, 380 nm, and photosynthetically active radiation (PAR) at our four exposure time periods (Table 1). Dosages from wavelengths 305–380 nm were combined to create a total UVR of ~0.66 kJ m^−2^/h. A potential consideration is that 66% of the radiation generated by the UVR bulb was Photosynthetically Active Radiation (PAR; ~1.92 kJ m^−2^/h), while 34% was UVR. Despite this dosage of PAR being low compared to environmental PAR, we cannot separate its effects from UVR in this experiment. Previous work has often included PAR during UVR treatments (Hite et al. 2016; Reguera et al. 2015), and as UVR is unlikely to occur without PAR in natural situations, it is likely that salamander larvae would elicit a response to PAR as a means of UVR protection (Blum and Valiñas 2022).

**TABLE 1:** Dosages (kJ/m^2^) of 305 nm, 320 nm, 380 nm, total UVR and PAR in kJ/m^−2^ for the three UVR exposure treatments (1, 4, 12 h). Values were calculated using a BIC logger (BIC2104RL‐UV).

Time/Wavelength	1 h	4 h	12 h
305 nm	0.1428	0.5712	1.7136
320 nm	0.48	1.92	5.76
380 nm	0.036	0.144	0.432
Total UVR	0.6588	2.6352	7.9056
PAR (400–700 nm)	1.9158	7.6632	22.9896

### 2.3 Pigmentation Measurements

**FIGURE 2:**
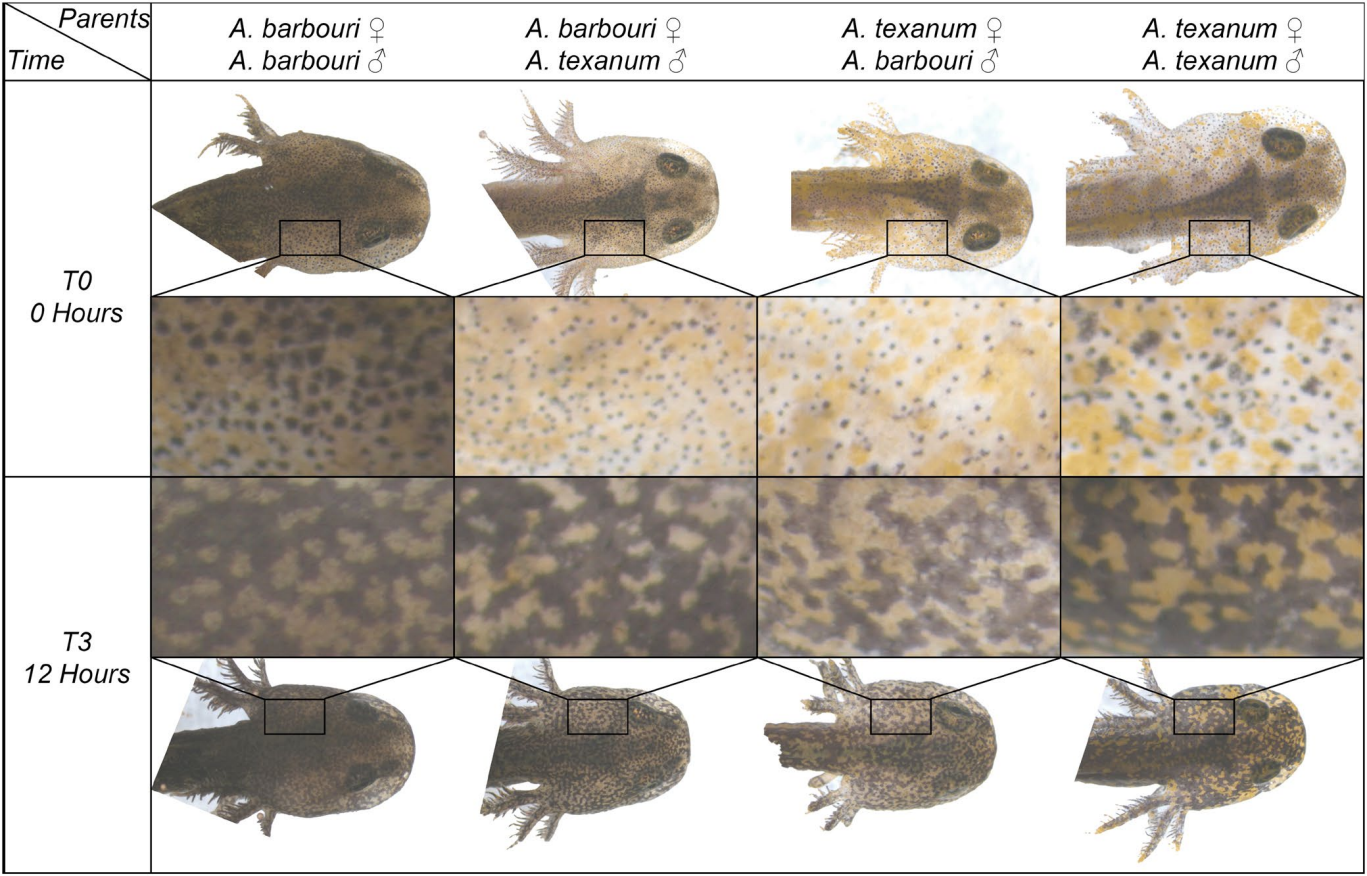
Comparison of *Ambystoma barbouri* and *A. texanum* larval melanophore and xanthophore responses with no UVR exposure and after 12 h of UVR exposure. 250 × 420‐pixel patches of head skin were used for analysis and are displayed here in the inlay.

We removed the larvae from their holding tubs 24 h prior to the experiment. We placed larvae, separated by genotype and clutch, into glass petri dishes filled with a few millimeters of water to just completely submerge the larvae. We covered the glass petri dishes with aluminum foil and placed them onto stainless steel sheets to prevent color response due to background color. We held the larvae in a dark environmental chamber at 15°C to prevent color response to temperature or light. We held larvae in these conditions for 24 h prior to UVR exposure (Garcia et al. 2003; Garcia and Sih 2003). After 24 h, we placed larvae under UVR light with a stainless‐steel background. Larvae were randomly assigned to UVR exposure treatments. The larvae were removed from UVR exposure after their treatment time was reached and were immediately taken for imaging. As reflectance spectra of eumelanin are well characterized (Vinod et al. 2024), and their visible reflectance is correlated to the quantity of melanin (Viator et al. 2004), we took visible spectrum photographs. We photographed larvae under LED lights at 10 ms of exposure and 9.8 times zoom. To standardize color change after experimental conditions, we photographed larvae in a dark room, only turning on the LED lights of the microscope immediately prior to photographing and held all other larvae under aluminum foil. Images were taken using the AxioCam MRc5 microscope with a Zeiss Discovery V12 and were exported as uncompressed BigTiff files and cropped into 250 × 420 pixel rectangles using Adobe Illustrator 2022 (Figure 2). To prevent biasing our estimates, cropped rectangles avoided the inclusion of areas in the center of the larval heads, which appear darker than their surroundings due to the presence of their brain.

*Ambystoma* salamanders have different pigmented cells, such as xanthophores and melanophores, which give them yellow and black/brown colors, respectively. We expected that their skin would get darker with increased UVR exposure, and that this change in color would be due to changes in the area covered by these pigmented cells, and that this response would also be dependent on genotype. Considering this, we performed two different measures of color change in response to UVR from a standard rectangle of 250 × 420 px extracted from one cheek of each larval picture. First, we transformed the cropped rectangle into grayscale and extracted the values of each pixel from 0 (white) to 1 (black), as a proxy of general skin darkness. Afterwards, from each standard rectangle, we measured the number of yellow (proxy of xanthophores), black (proxy of melanophores), and unpigmented pixels using the magic wand tool in Adobe Illustrator 2022. We set a magic wand threshold of 15%–20% and selected one color at a time. Once a color was selected, pixels selected were manually reduced or expanded to incorporate all pixels of the target color and to remove all other pixels from the selection. Because the Magic Wand tool selects pixels based on similarity to the initial pixel within the set threshold, and this can potentially influence the outcome, all pixel classification was conducted by the same trained individual using consistent visual criteria. While we did not formally quantify within‐image color variation or test sensitivity to initial pixel choice, using a single operator minimized inter‐observer bias and ensured consistent application of selection criteria across all images. Selected pixels were then counted using the histogram function and removed from the image. The same procedure was repeated for all remaining colors, and the total pixel count of each image was 105,000 pixels.

### 2.4 Statistical Analysis

Skin darkness as a response is a continuous variable, constrained between 0 and 1, which can be fitted using a Bayesian beta regression. We used a Bayesian model to estimate the effects on skin darkness that our three factors (time, mother genotype, father genotype) and the random effect of larval clutch had:

$$y\sim \text{Beta}\left(\mu ,\phi \right)$$

Where we model the mean of the distribution with a logit link following:

$$\text{logit}\left(\mu \right)=\beta_{\text{Time}}+\beta_{\text{Mother}}+\beta_{\text{Father}}+\beta_{\text{Mother}\times \text{Father}}+Z_{\text{Clutch}}$$

And the precision parameter of the distribution with a log‐link, following:

$$\log\left(\phi \right)=\gamma_{\text{Time}}+\gamma_{\text{Mother}}+\gamma_{\text{Father}}+\gamma_{\text{Mother}\times \text{Father}}+Z_{\text{Clutch}}$$

The values obtained as a proxy to pigmentation change can be considered compositional data, as we are measuring proportions of different colors or cells in a standard area that should sum to 1, or 100% of the area, in every instance (Van den Boogaart et al. 2021). Considering this, we used a Bayesian multilevel compositional data model and isometric log‐ratio transformations (ILR) of our data (Le et al. 2025). We defined two ILRs that we considered biologically relevant: the ratio of Yellow and Black pixels over Clear pixels, that is, the overall ratio of chromatophore cover across the skin; and the ratio between Black and Yellow pixels, that is, the ratio between melanophores and xanthophores. We included our three factors (time, mother genotype, and father genotype) as explanatory categorical variables and clutch as a random effect. This procedure models the joint distribution of the ILRs while keeping the constraints that all pixel proportions should sum to 100%, following (Le et al. 2025):

$$\mathrm{IL}R_{\left(\text{Yellow}+\text{Black}\right)/\text{Clear}},\mathrm{IL}R_{\text{Black}/\text{Yellow}}\sim \text{MVNormal}\left(\mu ,\sigma \right)$$

Where we model the means following:

$$\begin{array}{cc} \mu = & \beta_{\text{Time}}+\beta_{\text{Mother}}+\beta_{\text{Father}}+\beta_{\text{Mother}\times \text{Father}}\\ & +\beta_{\text{Mother}\times \text{Time}}+\beta_{\text{Father}\times \text{Time}}+Z_{\text{Clutch}} \end{array}$$

Given our three‐variable compositional response, ternary plots were used as the best representation for our compositional data (Van den Boogaart and Tolosana‐Delgado 2013). For both overall darkness and pigmentation composition models, we present results from the best models, selected among several more or less complex model definitions by using Watanabe‐Akaike Information Criterion (WAIC) and Leave‐One‐Out (LOO) cross validation, and ease of interpretation (see Material S1 and S3, respectively). Statistical analyses and plots were performed using the *brms*, *multilevelcoda*, *ggplot2*, and *ggtern* packages in R v4.4.1 (Bürkner 2017; Hamilton and Ferry 2018; Le and Wiley 2024; R Core Team 2024; Wickham 2016).

## 3 Results

**FIGURE 3:**
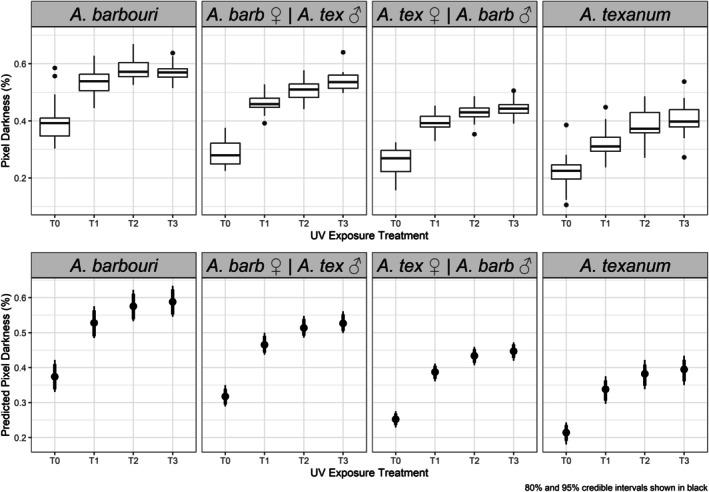
Observations (top) and Bayesian model predictions (bottom) of pixel darkness values in larvae from four different parental groupings of *A. barbouri* and *A. texanum* with increasing UVR exposure times.

**FIGURE 4:**
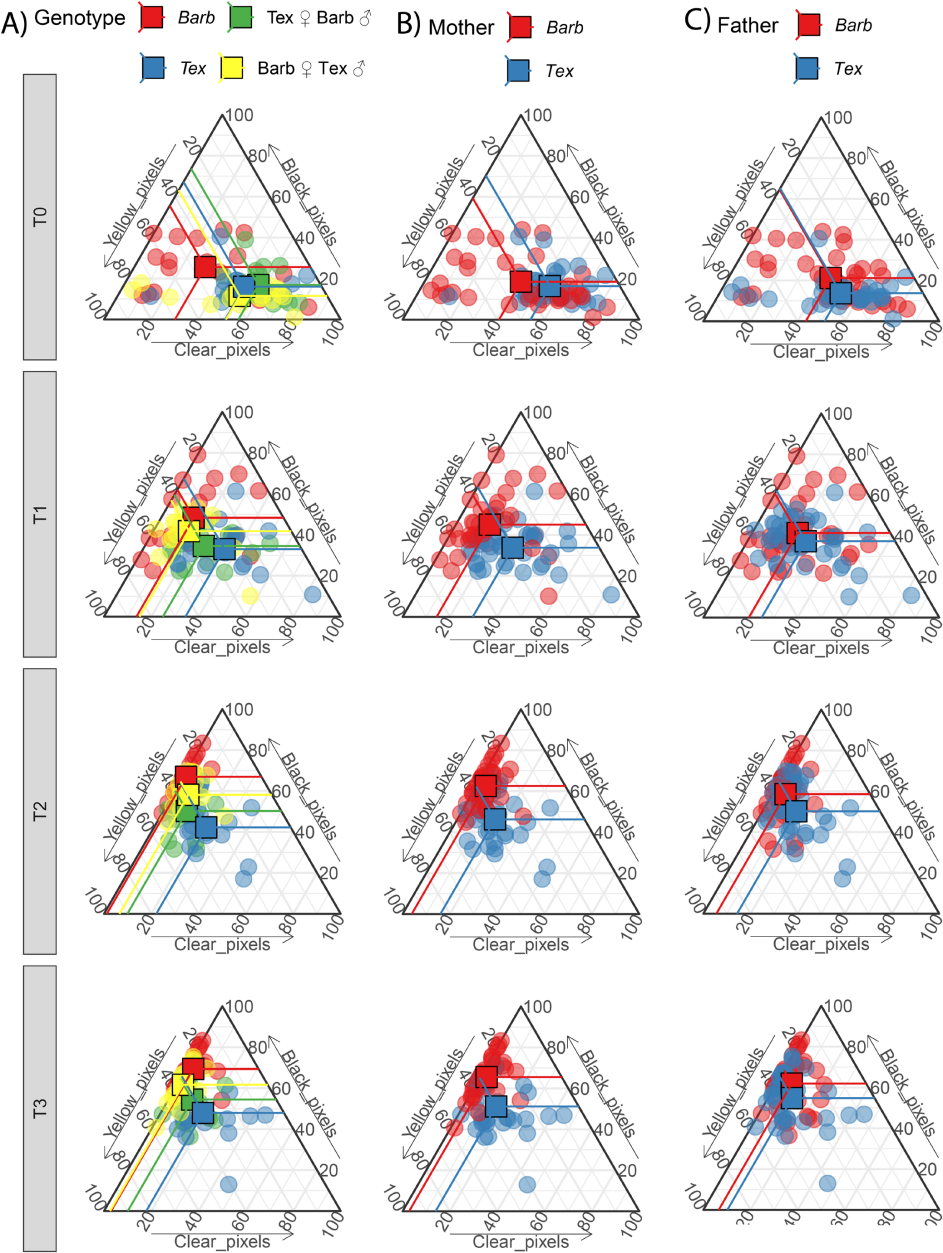
Compositional ternary plots showing chromatophore composition from larvae from four different parental groupings of *A. barbouri* and *A. texanum* with increasing UVR exposure times. Ternary plots indicate the proportion of color corresponding to Black, Yellow, and Clear pixels. Points close to the upper vertex of the triangle would represent fully (100%) black larvae; points close to the lower left vertex would represent 100% yellow larvae; and points close to the lower right vertex would represent 100% clear larvae. Therefore, points getting farther away from the lower right vertex (100% clear) and closer its opposite edge represent larvae increasing in black and/or yellow coloration as UVR exposure time increases. From left to right, each vertical set of panels displays change through UVR exposure time on samples (circles) and group means (squares) colored by: (A) the four genotype combinations, (B) the maternal lineage; and (C) the paternal lineage.

**FIGURE 5:**
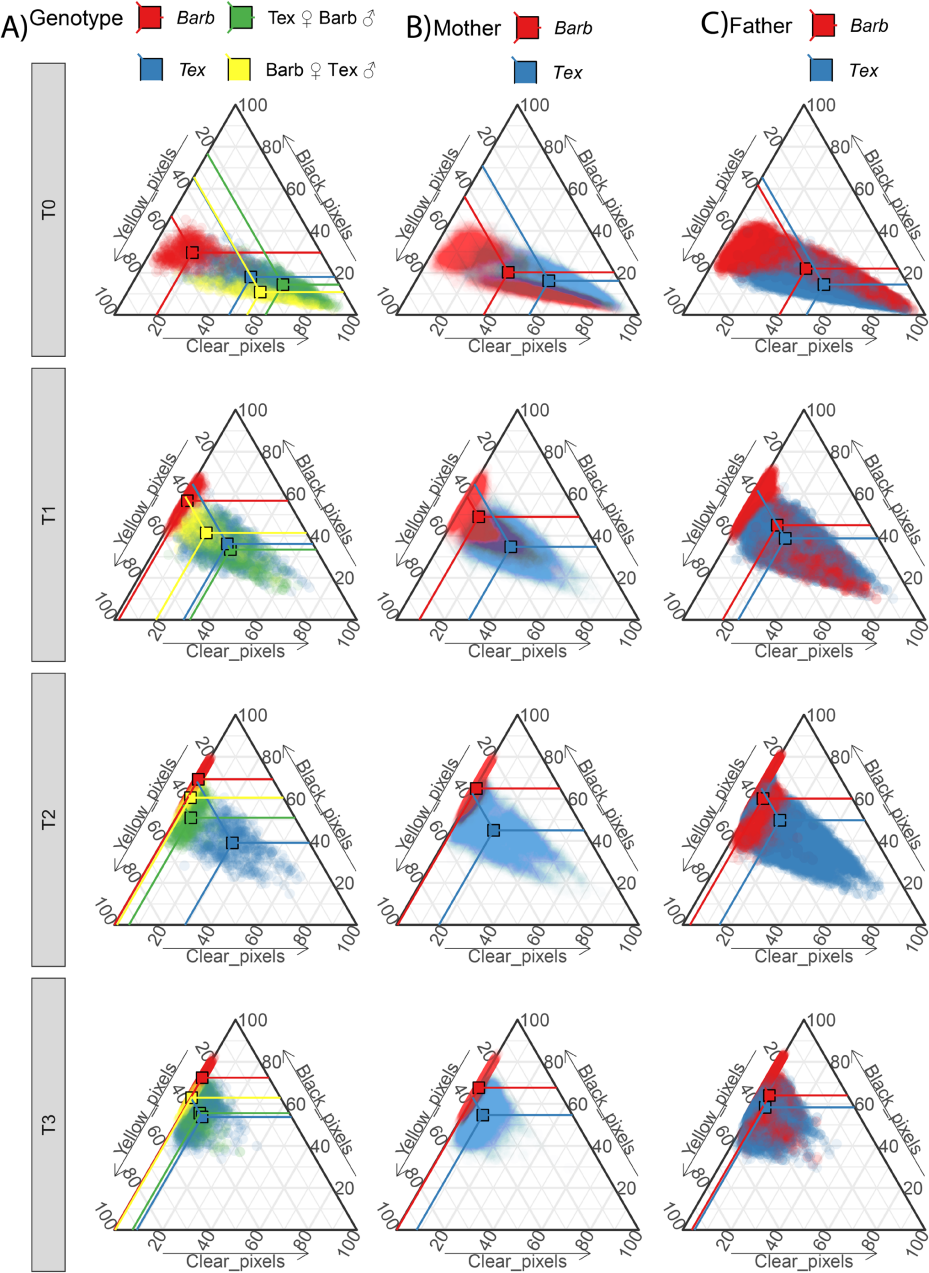
Compositional ternary plots showing predicted chromatophore composition from Bayesian predictive posterior distributions from four different parental groupings of *A. barbouri* and *A. texanum* with increasing UVR exposure times. Ternary plots indicate the proportion of color corresponding to Black, Yellow, and Clear pixels. Points close to the upper vertex of the triangle would represent fully (100%) black larvae; points close to the lower left vertex would represent 100% yellow larvae; and points close to the lower right vertex would represent 100% clear larvae. Model posterior means (squares) and predictive posterior samples (circles) corroborate our empirical results, with larvae getting darker (especially due to increasing black coloration) with increasing UVR exposure. From left to right, each vertical set of panels displays change through UVR exposure time in predictive posterior samples and posterior mean colored by: (A) the four genotype combinations, (B) the maternal lineage; and (C) the paternal lineage.

Hybrids of *A. texanum* and *A. barbouri* displayed intermediate pigmentation responses after UVR exposure when compared to both parental genotypes (Figures 3, 4, 5). These intermediate phenotypes tended to be closer to the phenotype displayed by the hybrid's maternal phenotype than that of their fathers. At baseline, *A. barbouri* had a darker pigmentation than *A. texanum* and both hybrid classes (Figure 3), mainly due to a greater area of the skin being covered by melanophores and xanthophores (Figures 4 and 5). After 4 h of UVR exposure, both darkness and pigment composition seemed to reach an asymptote or limit, with no significant differences when compared to the larval response after 12 h of UVR exposure. All treatments with one or more hours of UVR exposure, *A. barbouri* and *A. texanum* displayed the most extreme colorations, with the hybrids showing an intermediate phenotype being more similar to their maternal phenotypes. In addition to this, we evidenced that temperature increase due to light exposure does not have a significant effect on the change in larval darkness when compared to baseline (See Material S2: Figure S1).

In terms of overall darkness proportion, with 0 meaning completely white and 1 completely black, larval *A. barbouri* were darker than the other genotypes after any amount of UVR exposure, with an average darkness proportion of 0.528 (95% HDCI: 0.485–0.575) after 1 h, reaching 0.575 (95% HDCI: 0.534–0.622) after 4 h. *A. texanum* was found in the other extreme, with overall clearer coloration and darkness values of 0.338 (95% HDCI: 0.296–0.375) after 1 h of UVR exposure, reaching 0.382 (95% HDCI: 0.339–0.422) after 4 h. After 4 h, *A. barbouri ♀/A. texanum ♂* hybrids showed an average darkness proportion of 0.514 (95% HDCI: 0.486–0.548), while *A. texanum ♀/A. barbouri ♂* hybrids showed clearer coloration, with an average darkness of 0.434 (95% HDCI: 0.406–0.458). After 4 h of UVR exposure, having an *A. barbouri* mother made the larval darkness proportion increase by 36.5% (95% HDCI: 17.9%–55.1%), when compared to larvae with an *A. texanum* mother. Having an *A. barbouri* father also increased the expected proportion of skin darkness after 4 h of UVR exposure, though by a lower percentage of 13.9% (95% HDCI: −0.6% to 29.6%) when compared to larvae with an *A. texanum* father. Larval UVR responses seemed to reach an asymptote or limit after 4 h of UVR exposure in terms of darkness, with pure *A. barbouri* larvae reaching a limit close to (95% HDCI: 0.588 0.546–0.634) of pixel darkness, followed by hybrids with *A. barbouri* mother with 0.527 (95% HDCI: 0.499–0.561), hybrids with *A. texanum* mother with (95% HDCI: 0.477 0.420–0.472) and pure *A. texanum* larvae with 0.395 (95% HDCI: 0.350–0.434).

Regarding chromatophore cover, we observed that in all genotypes, increases in skin darkness were accompanied by compositional changes in the skin surface area occupied by melanophores and clear skin (Figures 4 and 5) while xanthophores remained somewhat constant. Ternary plots (Figure 4) can be read from left to right and top to bottom, with each vertical set of panels representing change through UVR exposure time in samples and group means based on: (A) the four genotype combinations; (B) the maternal lineage of each larva; and (C) the paternal lineage of each larva. It is noticeable in Figure 4A that from 1 to 12 h of UVR exposure hybrids show, on average, an intermediate color composition when compared to both non‐hybrid larvae. Furthermore, larvae color composition after UVR exposure was closer to that of their non‐hybrid maternal lineage counterparts. When comparing Figure 4B,C, we notice that after UVR exposure, larvae color composition clustered more in terms of their maternal rather than paternal lineages. Model estimates (Figure 5, Material S3) show that at baseline *A. barbouri* had a greater melanophore skin cover (29.7%, 95% HDCI: 20.3%–39.0%) when compared to *A. texanum* (18.0%, 95% HDCI: 8.1%–29.3%) and their hybrids. After 4 h of UVR exposure, melanophore cover increased, with *A. barbouri* still displaying the highest melanophore cover (69.2%, 95% HDCI: 58.6%–77.9%), with the hybrids showing intermediate phenotypes (*A. barbouri ♀*, 60.5%, 95% HDCI: 53.1–67.8 and *A. texanum ♀*, 51.0% 95% HDCI: 39.2%–64.0%) and *A. texanum* showing the lowest melanophore cover (39.2%, 95% HDCI: 24.4%–55.6%). On the other hand, xanthophore skin cover proportion seemed constrained between 30% and 40% regardless of genotype and UVR exposure time.

## 4 Discussion

As we hypothesized, *A. barbouri* larvae had a stronger UVR response, in terms of darkening and melanophore cover, than *A. texanum* ; while hybrids showed intermediate responses closer to their non‐hybrid maternal phenotype. We believe that these intermediate responses may reflect the presence of maternal effects from both species. Lastly, xanthophore proportion seemed to decrease or remain constant across all larval groups, corroborating that xanthophores may play a different role than UVR protection in these species.

Our results corroborate previous studies which evidenced that *A. barbouri* tends to have a darker coloration and UVR response when compared to *A. texanum* . Other studies further suggest that observed differences can result from different selective pressures that each species faces, such as increased UVR exposure and predation risk from stream predators in *A. barbouri* , and ephemerality of vernal pools in *A. texanum* (Garcia 2002; Garcia et al. 2003, 2004; Garcia and Sih 2003; Storfer et al. 1999). In general, it is considered that melanophores contribute to UVR protection, particularly from wavelengths that can cause genetic damage (Ahmed and Setlow 1993; Reinke et al. 2024); while lower melanin (or clearer coloration) can contribute to reducing predation risk (Polo‐Cavia and Gomez‐Mestre 2017; Reinke et al. 2024). Such a pattern aligns with the need of *A. barbouri* for enhanced UVR protection in its native streams, and the lack of UVR exposure and need for predator (such as larvae from dragonflies, conspecifics, and other *Ambystoma*) avoidance of *A. texanum* in their native breeding pools (Garcia and Sih 2003).

Building on these ecological differences, an additional consideration is the connection between UVR and its metabolic function, with melanin production being related to greater metabolic trade‐offs, diverting energy to this activity rather than to growth or reproduction (S. Britton and Davidowitz 2023; Polo‐Cavia and Gomez‐Mestre 2017). That said, ultraviolet radiation, particularly at low levels, has been shown to play a beneficial role in promoting growth and supporting metabolic function in amphibians (Alton et al. 2012; Formicki et al. 2003).

Interestingly, the mitochondrial genomes of *A. barbouri* and *A. texanum* remain strongly associated with their respective habitats despite extensive evidence of hybridization (Bi and Bogart 2010; Denton et al. 2014; Fitzgerald 2023; Kraus and Petranka 1989). Given that *A. texanum* often occupies shaded environments with low UVR exposure, it is possible that this species benefits from allowing some UVR penetration when it does occur. Rather than completely blocking UVR exposure, a reduced number of melanophores could allow for limited UVR absorption, facilitating essential metabolic activity. Since melanophores do not multiply in response to UVR but instead distribute melanin through intracellular organelle spreading, this mechanism may represent an adaptive balance between UVR protection and metabolic necessity in shaded environments.

Similar trade‐offs between crypsis, chromatophore cover, and UVR protection, linked to metabolic costs and predation risks, have been previously documented across taxa (Alfakih et al. 2022); for example, trade‐offs between cryptic coloration and brain size (Liao et al. 2022) or enhanced UVR protection with decreased chytrid fungus immunity in frogs (Cramp et al. 2022), and octopus chromatophore expansion/contraction and depletion of its energy reserves (Sonner and Onthank 2024). Previous literature shows that when species are faced with such trade‐offs, naturally occurring hybrids can present hybrid vigor or inferiority, which may reduce or reinforce postzygotic reproductive barriers between species (Porretta and Canestrelli 2023). This highlights how plastic and adaptive responses to environmental gradients can influence fitness and potentially shape species boundaries in hybrid zones.

In the case of *A. barbouri* and *A. texanum* , we initially hypothesized that hybrids exhibiting UVR responses similar to or exceeding those of their parent species could potentially overcome some of the trade‐offs related to melanin production and UVR protection. To us, this meant displaying a darkening as great as *A. barbouri* after UVR exposure and being as clear as *A. texanum* before UVR exposure. Our data showed that hybrids resembled the *A. texanum* phenotype in terms of darkening and color ratios before UVR exposure; however, they displayed intermediate phenotypes across the 1–12 h of UVR exposure. Our results suggest that hybrids could be less protected from UVR than *A. barbouri* when exposed to the same breeding habitat and selective pressures. While our results demonstrate these intermediate pigmentation patterns, we did not directly assess fitness outcomes; therefore, any implications for UVR protection or adaptation should be investigated in future studies. Additionally, because our imaging protocol required briefly exposing larvae to a brief period of darkness before photographing, we recognize a potential source of bias. Specifically, if camouflage‐induced darkening occurs in response to dark conditions and is aligned with the strong UVR‐response phenotype, then individuals with a darker baseline (e.g., *A. barbouri* ) may show less further change than lighter individuals (e.g., *A. texanum* ) (Liedtke et al. 2023). This could bias apparent differences in plasticity between groups, particularly at later time points. This possibility highlights the importance of disentangling baseline melanophore state, camouflage signaling, and UVR‐induced pigment dynamics in future research.

Notably, the observed hybrid intermediate phenotypes appeared closer to their maternal phenotypes, consistent with potential maternal effects. Aside from UVR response, such maternal effects have also been observed in other traits relevant to the hybridization of these species (Fitzgerald 2023), including survival to metamorphosis. Maternal effects are being increasingly recognized as powerful drivers of hybrid outcomes, with potential applications in conservation (Chan et al. 2021; Gallego‐Tévar et al. 2019), agriculture (Antony John et al. 2024; Aycan et al. 2021; Debes et al. 2013), and our general understanding of species evolution (Moore et al. 2019; Videvall et al. 2016). Furthermore, these maternal effects can influence hybrid zone dynamics and affect the degree of reproductive isolation between populations by giving hybrid individuals increased persistence in parental habitats (Gallego‐Tévar et al. 2019).

Maternal effects can occur due to mitochondrial inheritance, sex chromosome linked traits, epigenetic changes, and maternal yolk provisioning (Chan et al. 2021; Kim et al. 2022). Sex‐linked traits are also unlikely to explain our results, as larvae from different clutches within the same parental pairings showed consistent UVR responses, without patterns suggesting sex‐specific differences. Sex determination in *Ambystoma* salamanders follows a ZZ/ZW mechanism, in which females are the heterogametic sex (Smith and Voss 2009). If UVR response was a sex‐linked trait, we would expect greater variability within each clutch due to random sampling of male and female larvae (assuming a roughly 1:1 sex ratio), but this was not the case. This can be tested empirically in future experiments by determining larvae sex and including sex as a factor within the models. Under the assumption that observed changes in UVR response relate to melanin synthesis, mitochondrial inheritance is also unlikely, as genes related to melanin formation and storage in amphibians, such as TYR, TYRP1, PMEL, OCA2, and Mc1r, are nuclear genes (Burgon et al. 2020; Cecil et al. 2024; Deng et al. 2023; Woodcock et al. 2017). That leaves the options of different mitochondrial genes influencing UVR response, inherited epigenetic changes from the mothers, and developmental effects provided by the mother's yolk, as previous studies showed that mothers can provide the yolk with carotenoids or other substances that help during development (Byrne and Silla 2017; Muller et al. 2012). Further research is needed to determine the extent and persistence of these maternal effects within and across multiple generations.

Lastly, our study showed that xanthophore skin cover did not significantly change, likely not contributing to the darkening of the larvae as UVR exposure increased. The observed lack of change in xanthophore cover suggests that these cells' primary function is not in UVR protection, though this does not mean they are not involved in the process. Current literature indicates that xanthophores may be important for camouflage, social signaling, thermoregulation, and UVR protection (Blount and McGraw 2008; Gould and McHenry 2024). Even if xanthophores provide a baseline level of protection that does not change with the time of UVR exposure, this level of protection does not seem to be determined by the maternal effects discussed above.

In summary, our findings demonstrate that *Ambystoma barbouri* larvae show a stronger darkening response and greater melanophore coverage under UVR exposure compared to *A. texanum* , with hybrids exhibiting intermediate phenotypes that more closely resemble their maternal lineage. These results are consistent with differences in natural habitat UVR exposure and suggest that maternal effects may influence larval pigmentation responses. However, as our study did not measure fitness‐related outcomes associated with these pigmentation patterns, we cannot directly determine whether intermediate hybrid phenotypes are maladaptive, that is, contributing to reproductive isolation. Given that these salamanders have long lifespans and do not reach sexual maturity until 2–3 years, the long‐term implications of intermediate pigmentation in hybrids, including potential effects on fitness or gene flow across generations, remain unknown. Further work assessing post‐F1 and F2 generations and measuring fitness‐related outcomes needs to be done to determine whether hybridization poses a risk for the persistence of endangered *A. barbouri* populations. Taken together, these findings contribute to a growing understanding of how environmental factors can shape hybrid phenotypes, potentially influencing hybrid fitness and the formation or maintenance of reproductive barriers across taxa.

## Author Contributions

**Alfredo Ascanio:** conceptualization (equal), data curation (equal), formal analysis (equal), investigation (equal), methodology (lead), software (lead), visualization (lead), writing – original draft (equal), writing – review and editing (lead). **Victor Fitzgerald:** conceptualization (equal), data curation (equal), investigation (lead), methodology (equal), project administration (lead), software (supporting), supervision (equal), validation (equal), writing – original draft (equal), writing – review and editing (supporting). **Patrick Altomari:** data curation (equal), investigation (supporting), software (supporting), writing – original draft (supporting). **Jason T. Bracken:** conceptualization (supporting), data curation (supporting), formal analysis (supporting), investigation (supporting), validation (supporting), writing – original draft (supporting), writing – review and editing (supporting). **Tereza Jezkova:** conceptualization (supporting), data curation (supporting), funding acquisition (lead), investigation (supporting), project administration (supporting), supervision (lead), writing – original draft (supporting), writing – review and editing (supporting).

## Ethics Statement

Miami University IACUC 827: “Interactive effects of contaminants, invasive species, pathogens, and habitat manipulation on amphibian populations and community structure: How multiple stressors may contribute to amphibian declines & influence community dynamics”. Ohio DNR, Division of Wildlife, Wild Animal Permit #22‐014. Indiana DNR, Division of Fish &Wildlife, Scientific Purposes License #3167. Kentucky Department of Fish & Wildlife Resources, Educational Wildlife Collecting #SC111129.

## Conflicts of Interest

The authors declare no conflicts of interest.

## Supporting information


**Appendix S1:** ece371911‐sup‐0001‐AppendixS1.docx.

## Data Availability

Data and code are freely available at the Zenodo repository: https://doi.org/10.5281/zenodo.15411414.
