# Supplementary material for: Larval Pigmentation Reveals Environmental and Genetic Influences in Hybridizing Ambystoma Salamanders
Source: Ecol Evol. 2025 Aug 3;15(8):e71911. doi: 10.1002/ece3.71911 (PMC12318607; doi:10.1002/ece3.71911)
Supplement: Supplementary file 1 — Appendix S1: ece371911‐sup‐0001‐AppendixS1.docx. [file ECE3-15-e71911-s001.docx]

Ecology and Evolution – Research Article

**Title: Larval Pigmentation Reveals Environmental and Genetic Influences in Hybridizing Ambystoma Salamanders**

Running title: Hybridization and Pigmentation in Ambystoma

Contents

[Bayesian Beta Regression Models 2](#_Toc202307930)

[Model Comparisons: 2](#_Toc202307931)

[Model 2 (Best Model) 2](#_Toc202307932)

[Model 1 3](#_Toc202307933)

[Model 2 (Best Model) Coefficients 3](#_Toc202307934)

[Model 3 3](#_Toc202307935)

[Model 4 4](#_Toc202307936)

[Model 5 4](#_Toc202307937)

[Larval Darkening Compared to Temperature (no UVR exposure) 4h treatment 5](#_Toc202307938)

[Figure S1. 5](#_Toc202307939)

[Model Definition and Coefficients 5](#_Toc202307940)

[Bayesian Multilevel CoDA Models 7](#_Toc202307941)

[Shared Features Across Models 7](#_Toc202307942)

[Model Comparisons: 7](#_Toc202307943)

[Model 3 (Best Model) 7](#_Toc202307944)

[Model 3 Coefficients (Best Model) 8](#_Toc202307945)

[Model 1 9](#_Toc202307946)

[Model 2 9](#_Toc202307947)

[Model 4 9](#_Toc202307948)

[Model 5 9](#_Toc202307949)

[Model 6 9](#_Toc202307950)

# Supplementary Material S1: Bayesian Beta Regression Models

Response follows a beta distribution with parameters *a* and *b*. This definition can be reparametrized in terms of $\mu$ (mean) and $\phi$ (precision), which aid with interpretation (see reparameterization below)

$$f\left( y_{i} \mid a,b \right)=\frac{y_{i}^{a-1}\left( 1-y_{i} \right)^{b-1}}{B\left( a,b \right)}$$

$$a = \mu\cdot\phi$$

$$b=\left( 1-\mu\right)\phi$$

$$f\left( y_{i} \mid\mu,\phi\right)=\frac{y_{i}^{\mu\phi-1}\left( 1-y_{i} \right)^{\left( 1-\mu\right)\phi-1}}{B\left( \mu\phi,\left( 1-\mu\right)\phi\right)}$$

Mean submodels use a logit-link on mu.
Precision submodels use a log-link log(phi).
Random intercept for Clutch (Z_Clutch) included in both.
Default *brms* priors were used

## Model Comparisons:

|  | LOO_elpd_diff | LOO_se_diff | WAIC_elpd_diff | WAIC_se_diff |
| --- | --- | --- | --- | --- |
| m1 | 0 | 0 | 0 | 0 |
| **m2** | **-0.2** | **0.7** | **-0.2** | **0.7** |
| m4 | -2.7 | 5.1 | -1.8 | 4.9 |
| m3 | -3.4 | 5.9 | -1.7 | 5.6 |
| m5 | -3.4 | 5.9 | -1.7 | 5.6 |

## Model 2 (Best Model)

Mean submodel (logit link):

$$\text{logit}\left( \mu\right)=\beta_{\text{Time}}+\beta_{\text{Mother}}+\beta_{\text{Father}}+\beta_{\text{Mother}\times\text{Father}}+Z_{\text{Clutch}}$$

Precision submodel:

$$\log\left( \phi\right)=\gamma_{\text{Time}}+\gamma_{\text{Mother}}+\gamma_{\text{Father}}+\gamma_{\text{Mother}\times\text{Father}}+Z_{\text{Clutch}}$$

##

## Model 1

Mean submodel (logit link):

$$\text{logit}\left( \mu\right)=\beta_{\text{Time}}+\beta_{\text{Mother}}+\beta_{\text{Father}}+Z_{\text{Clutch}}$$

Precision submodel:

$$\log\left( \phi\right)=\gamma_{\text{Time}}+\gamma_{\text{Mother}}+\gamma_{\text{Father}}+Z_{\text{Clutch}}$$

## Model 2 (Best Model) Coefficients

| Coefficients | Estimate | Est.Error | l-95% CI | u-95% CI | Rhat | Bulk_ESS | Tail_ESS |
| --- | --- | --- | --- | --- | --- | --- | --- |
| TimeT0 | -0.521 | 0.071 | -0.665 | -0.374 | 1.001 | 1439.615 | 1689.622 |
| TimeT1 | 0.101 | 0.066 | -0.03 | 0.238 | 1.001 | 1359.015 | 1440.863 |
| TimeT2 | 0.298 | 0.065 | 0.169 | 0.433 | 1.001 | 1266.532 | 1581.019 |
| TimeT3 | 0.347 | 0.066 | 0.212 | 0.481 | 1.001 | 1323.33 | 1536.048 |
| MotherTex | -0.555 | 0.073 | -0.701 | -0.41 | 1.002 | 1581.295 | 1912.838 |
| FatherTex | -0.233 | 0.075 | -0.383 | -0.088 | 1.002 | 1797.831 | 2062.05 |
| phi_TimeT0 | 4.509 | 0.345 | 3.821 | 5.226 | 1.001 | 2103.294 | 1910.647 |
| phi_TimeT1 | 5.486 | 0.355 | 4.789 | 6.174 | 1 | 2111.8 | 1898.751 |
| phi_TimeT2 | 5.6 | 0.359 | 4.874 | 6.291 | 1.001 | 2083.343 | 2166.035 |
| phi_TimeT3 | 5.613 | 0.35 | 4.902 | 6.314 | 1 | 2144.293 | 1987.505 |
| phi_MotherTex | -0.289 | 0.359 | -0.983 | 0.449 | 1.002 | 2393.983 | 2285.986 |
| phi_FatherTex | -0.432 | 0.371 | -1.138 | 0.345 | 1 | 1996.135 | 1887.814 |
| sd(Intercept) | 0.114 | 0.038 | 0.063 | 0.21 | 1.002 | 1210.532 | 1833.443 |
| sd(phi_Intercept) | 0.52 | 0.206 | 0.206 | 1 | 1.001 | 1343.929 | 2066.423 |

## Model 3

Mean submodel (logit link):

$$\text{logit}\left( \mu\right)=\beta_{\text{Time}}+\beta_{\text{Mother}}+\beta_{\text{Father}}+\beta_{\text{Mother}\times\text{Father}\times\text{Time}}+Z_{\text{Clutch}}$$

Precision submodel:

$$\log\left( \phi\right)=\gamma_{\text{Time}}+\gamma_{\text{Mother}}+\gamma_{\text{Father}}+\gamma_{\text{Mother}\times\text{Father}\times\text{Time}}+Z_{\text{Clutch}}$$

## Model 4

Mean submodel (logit link):

$$\text{logit}\left( \mu\right)=\beta_{\text{Time}}+\beta_{\text{Mother}}+\beta_{\text{Father}}+\beta_{\text{Mother}\times\text{Time}}+\beta_{\text{Father}\times\text{Time}}+Z_{\text{Clutch}}$$

Precision submodel:

$$\log\left( \phi\right)=\gamma_{\text{Time}}+\gamma_{\text{Mother}}+\gamma_{\text{Father}}+\gamma_{\text{Mother}\times\text{Time}}+\gamma_{\text{Father}\times\text{Time}}+Z_{\text{Clutch}}$$

## Model 5

Mean submodel (logit link):

$$\text{logit}\left( \mu\right)=\beta_{\text{Time}}+\beta_{\text{Mother}}+\beta_{\text{Father}}+\beta_{\text{Mother}\times\text{Father}}+\beta_{\text{Mother}\times\text{Time}}+\beta_{\text{Father}\times\text{Time}}+\beta_{\text{Mother}\times\text{Father}\times\text{Time}}+Z_{\text{Clutch}}$$

Precision submodel:

$$\log\left( \phi\right)=\gamma_{\text{Time}}+\gamma_{\text{Mother}}+\gamma_{\text{Father}}+\gamma_{\text{Mother}\times\text{Father}}+\gamma_{\text{Mother}\times\text{Time}}+\gamma_{\text{Father}\times\text{Time}}+\gamma_{\text{Mother}\times\text{Father}\times\text{Time}}+Z_{\text{Clutch}}$$

# Supplementary Material S2: Larval Darkening Compared to Temperature (no UVR exposure) 4h treatment


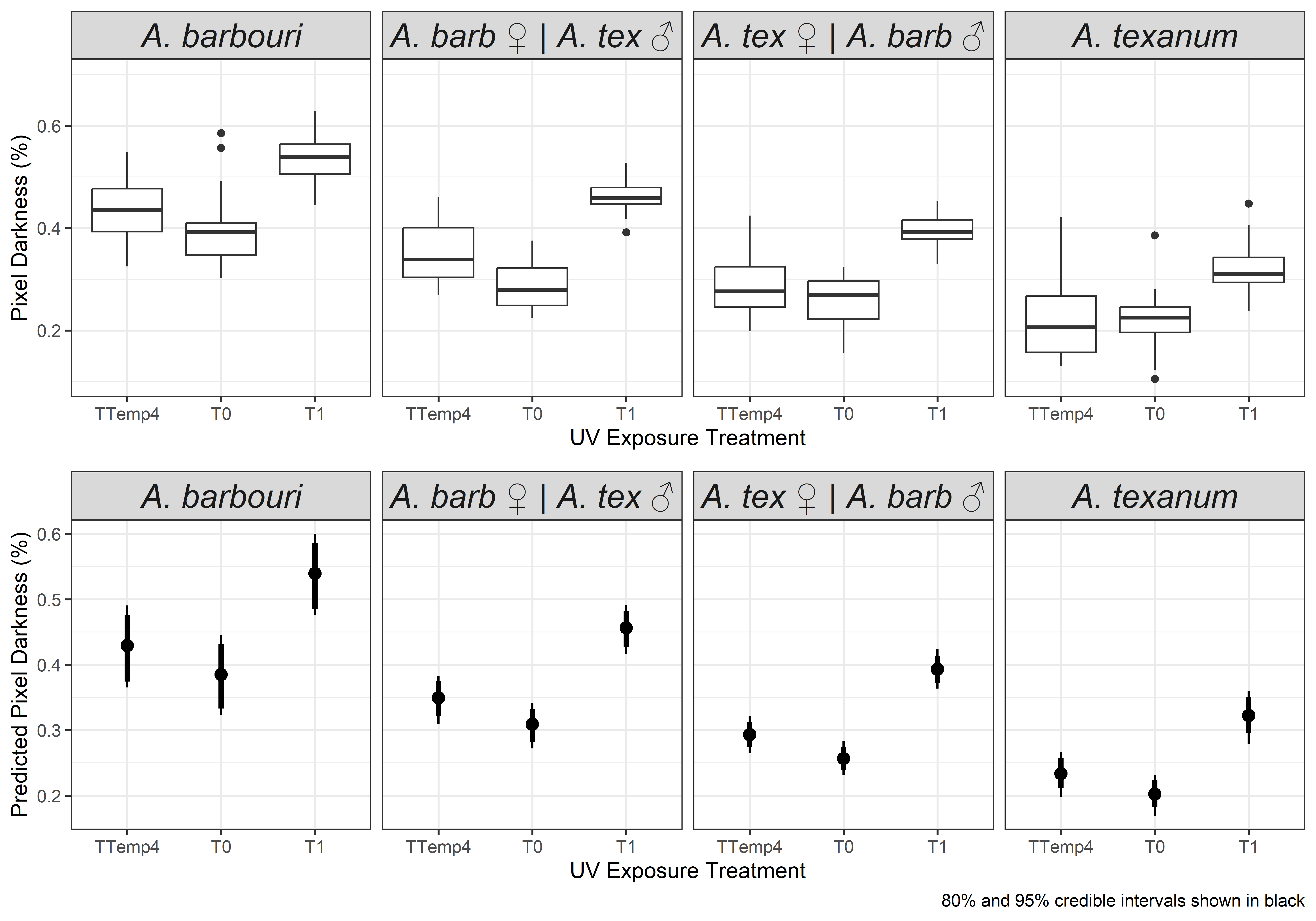


## Figure S1: Observations (top) and Bayesian model predictions (bottom) of pixel darkness values in larvae from four different parental groupings of *A. barbouri* and *A. texanum*, comparing the effect of 4 hours of temperature exposure in darkness (TTemp4) with 4 hours of UVR exposure (T1) and 0 hours of exposure to temperature and UVR (T0).

## Model Definition and Coefficients

Mean submodel (logit link):

$$\text{logit}\left( \mu\right)=\beta_{\text{Time}}+\beta_{\text{Mother}}+\beta_{\text{Father}}+\beta_{\text{Mother}\times\text{Father}}+Z_{\text{Clutch}}$$

Precision submodel:

$$\log\left( \phi\right)=\gamma_{\text{Time}}+\gamma_{\text{Mother}}+\gamma_{\text{Father}}+\gamma_{\text{Mother}\times\text{Father}}+Z_{\text{Clutch}}$$

| Coefficients | Estimate | Est.Error | l-95% CI | u-95% CI | Rhat | Bulk_ESS | Tail_ESS |
| --- | --- | --- | --- | --- | --- | --- | --- |
| TimeTTemp4 | -0.29 | 0.1 | -0.49 | -0.1 | 1 | 1408 | 1733 |
| TimeT0 | -0.47 | 0.1 | -0.67 | -0.28 | 1 | 1370 | 1720 |
| TimeT1 | 0.16 | 0.09 | -0.03 | 0.34 | 1 | 1311 | 1656 |
| MotherTex | -0.59 | 0.13 | -0.86 | -0.32 | 1 | 1377 | 1495 |
| FatherTex | -0.33 | 0.13 | -0.6 | -0.07 | 1 | 1299 | 1813 |
| MotherTex:FatherTex | 0.02 | 0.19 | -0.36 | 0.43 | 1 | 1349 | 1601 |
| phi_TimeTTemp4 | 4.32 | 0.33 | 3.66 | 4.96 | 1.01 | 1379 | 1205 |
| phi_TimeT0 | 4.43 | 0.31 | 3.82 | 5.04 | 1 | 1361 | 825 |
| phi_TimeT1 | 5.37 | 0.33 | 4.72 | 6.01 | 1 | 1505 | 1496 |
| phi_MotherTex | -0.25 | 0.39 | -1.03 | 0.52 | 1 | 1281 | 782 |
| phi_FatherTex | -0.02 | 0.41 | -0.84 | 0.75 | 1 | 1272 | 1365 |
| phi_MotherTex:FatherTex | -0.53 | 0.57 | -1.64 | 0.62 | 1 | 1572 | 1017 |
| sd(Intercept) | 0.15 | 0.05 | 0.08 | 0.28 | 1 | 1540 | 2054 |
| sd(phi_Intercept) | 0.28 | 0.2 | 0.01 | 0.74 | 1 | 1253 | 1816 |

# Supplementary Material S3: Bayesian Multilevel CoDA Models

## Shared Features Across Models

Response: Two ILR coordinates (ilr1, ilr2) modeled jointly using the following Sequential Binary Partitions (SBP):

$$\text{ILR}_{1,2}\sim MVNormal\left( \mu,\sigma\right)$$

| Pixel Ratio Partitions | Yellow | Black | Clear |
| --- | --- | --- | --- |
| ILR1 $[\left( Yellow+Black \right)/Clear]$ | 1 | 1 | -1 |
| ILR2 $(Black/Yellow)$ | -1 | 1 | 0 |

Random Effects: Clutch-specific intercepts (Z_Clutch).
Data: Compositional pigment proportions transformed via ILR.

## Model Comparisons:

|  | LOO_elpd_diff | LOO_se_diff |
| --- | --- | --- |
| **m3** | **0** | **0** |
| m4 | -1.8 | 0 |
| m5 | -3.9 | 1.3 |
| m6 | -4.2 | 1.8 |
| m2 | -21.2 | -2.6 |
| m1 | -23.1 | -2.8 |

## Model 3 (Best Model)

Mean submodel for ILR coordinates:

$$\mu= \beta_{\text{Time}}+\beta_{\text{Mother}}+\beta_{\text{Father}}+\beta_{\text{Mother}\times\text{Father}}+\beta_{\text{Mother}\times\text{Time}}+\beta_{\text{Father}\times\text{Time}}+Z_{\text{Clutch}}$$

## Model 3 Coefficients (Best Model)

| Coefficient | Estimate | Est.Error | l-95% CI | u-95% CI | Rhat | Bulk_ESS | Tail_ESS |
| --- | --- | --- | --- | --- | --- | --- | --- |
| ilr1_Intercept | 0.77 | 0.48 | -0.18 | 1.71 | 1 | 1861 | 2392 |
| ilr2_Intercept | -0.42 | 0.17 | -0.78 | -0.08 | 1 | 1577 | 1932 |
| ilr1_TimeT1 | 2.08 | 0.59 | 0.92 | 3.25 | 1 | 2154 | 2733 |
| ilr1_TimeT2 | 6.14 | 0.58 | 5 | 7.27 | 1 | 2190 | 2538 |
| ilr1_TimeT3 | 5.59 | 0.58 | 4.43 | 6.71 | 1 | 2190 | 2733 |
| ilr1_MotherT | -1.81 | 0.62 | -3 | -0.58 | 1 | 1930 | 2445 |
| ilr1_FatherT | -1.63 | 0.63 | -2.81 | -0.35 | 1 | 1897 | 2301 |
| ilr1_MotherT:FatherT | 2.14 | 0.65 | 0.86 | 3.4 | 1 | 2179 | 2072 |
| ilr1_TimeT1:MotherT | -0.91 | 0.66 | -2.23 | 0.37 | 1 | 2351 | 2725 |
| ilr1_TimeT2:MotherT | -3.32 | 0.66 | -4.63 | -2.05 | 1 | 2620 | 2962 |
| ilr1_TimeT3:MotherT | -2.97 | 0.67 | -4.28 | -1.71 | 1 | 2578 | 2899 |
| ilr1_TimeT1:FatherT | -0.41 | 0.69 | -1.79 | 0.92 | 1 | 2440 | 2483 |
| ilr1_TimeT2:FatherT | -2.1 | 0.69 | -3.49 | -0.76 | 1 | 2381 | 2463 |
| ilr1_TimeT3:FatherT | -0.71 | 0.68 | -2.07 | 0.63 | 1 | 2578 | 2949 |
| ilr2_TimeT1 | 0.64 | 0.11 | 0.42 | 0.85 | 1 | 2190 | 2853 |
| ilr2_TimeT2 | 1 | 0.11 | 0.78 | 1.22 | 1 | 2318 | 2648 |
| ilr2_TimeT3 | 1.1 | 0.11 | 0.89 | 1.32 | 1 | 2308 | 2972 |
| ilr2_MotherT | 0.07 | 0.25 | -0.42 | 0.57 | 1.01 | 1329 | 1491 |
| ilr2_FatherT | -0.4 | 0.25 | -0.89 | 0.1 | 1 | 1493 | 1467 |
| ilr2_MotherT:FatherT | 0.29 | 0.34 | -0.42 | 0.97 | 1 | 1400 | 1307 |
| ilr2_TimeT1:MotherT | -0.33 | 0.13 | -0.59 | -0.08 | 1 | 3064 | 2991 |
| ilr2_TimeT2:MotherT | -0.52 | 0.13 | -0.78 | -0.28 | 1 | 3071 | 2988 |
| ilr2_TimeT3:MotherT | -0.46 | 0.13 | -0.71 | -0.21 | 1 | 3090 | 2946 |
| ilr2_TimeT1:FatherT | 0.18 | 0.13 | -0.07 | 0.42 | 1 | 2860 | 2738 |
| ilr2_TimeT2:FatherT | 0.14 | 0.13 | -0.11 | 0.38 | 1 | 2799 | 3100 |
| ilr2_TimeT3:FatherT | 0.09 | 0.13 | -0.16 | 0.34 | 1 | 2725 | 3050 |
| sd(ilr1_Intercept) | 0.31 | 0.22 | 0.02 | 0.84 | 1 | 1245 | 1520 |
| sd(ilr2_Intercept) | 0.25 | 0.1 | 0.13 | 0.48 | 1 | 1110 | 1588 |
| sigma_ilr1 | 2 | 0.09 | 1.84 | 2.18 | 1 | 5261 | 3088 |
| sigma_ilr2 | 0.39 | 0.02 | 0.35 | 0.42 | 1 | 4686 | 2649 |
| rescor(ilr1,ilr2) | -0.03 | 0.06 | -0.14 | 0.09 | 1 | 4975 | 2850 |

## Model 1

Mean submodel for ILR coordinates:

$$\mu= \beta_{\text{Time}}+\beta_{\text{Mother}}+\beta_{\text{Father}}+Z_{\text{Clutch}}$$

## Model 2

Mean submodel for ILR coordinates:

$$\mu= \beta_{\text{Time}}+\beta_{\text{Mother}}+\beta_{\text{Father}}+\beta_{\text{Mother}\times\text{Father}}+Z_{\text{Clutch}}$$

## Model 4

Mean submodel for ILR coordinates:

$$\mu= \beta_{\text{Time}}+\beta_{\text{Mother}}+\beta_{\text{Father}}+\beta_{\text{Mother}\times\text{Time}}+\beta_{\text{Father}\times\text{Time}}+Z_{\text{Clutch}}$$

## Model 5

Mean submodel for ILR coordinates:

$$\mu=\beta_{\text{Time}}+\beta_{\text{Mother}}+\beta_{\text{Father}}+\beta_{\text{Mother}\times\text{Father}}+\beta_{\text{Mother}\times\text{Time}}+\beta_{\text{Father}\times\text{Time}}+\beta_{\text{Mother}\times\text{Father}\times\text{Time}}+Z_{\text{Clutch}}$$

## Model 6

Mean submodel for ILR coordinates:

$$\mu=\beta_{\text{Time}}+\beta_{\text{Mother}}+\beta_{\text{Father}}+\beta_{\text{Mother}\times\text{Father}\times\text{Time}}+Z_{\text{Clutch}}$$

# References
